# Society Meetings

**Published:** 1917-12

**Authors:** 


### SOCIETY MEETINGS

---

Brief reports and announcements of meetings of societies of Western New York. and those of general scope, are requested from Secretaries. Copy should be on hand the fifteenth of the month. Full transactions will be published at cost of composition.

---

**DOCTOR: Is your Society properly represented here? If not, please bring our standing announcement to the attention of the members.**

---

**The Buffalo Academy of Medicine,** Section of Pathology, October 24. Program: Organization of Work of the Laboratories of the British Medical Service in the Field, Col. George G. Nasmith, C. A. M. S. of Toronto.

Section of Surgery, November 7. Program: Some Complications and Incorrect Diagnoses in Urological Surgery, Dr. Oswald S. Lowsley, New York; Some Practical Points about Renal Infection.

Section of Medicine, November 14. Program: Laboratory Diagnosis and Intravenous Therapy in Typhoid Fever, Dr. Barton F. Hauenstein; Studies of Salt in Nephritis, Dr. Byron D. Bowen.

---

**Rochester Academy of Medicine.** Section III, November 14. Program: The Prenatal care of Pregnant Women, Dr. J. T. Beirmeister.

---

**Elmira Academy of Medicine.** November 7. Program: The Surgeon's Attitude Toward Painful Lumps in Breast, Dr. A. W. Booth; Chronic Nephritis, Dr. Maurice Packard, New York.

